# Target Contour Recovering for Tracking People in Complex Environments

**DOI:** 10.1155/2012/506908

**Published:** 2011-10-24

**Authors:** Jianhua Zhang, Sheng Liu, Y. F. Li, Jianwei Zhang

**Affiliations:** ^1^Group TAMS, Department of Informatics, University of Hamburg, 22527 Hamburg, Germany; ^2^College of Information Engineering, Zhejiang University of Technology, 18 Chaowang Road, Hangzhou 310014, China; ^3^Department of System Engineering and Engineering Management, City University of Hong Kong, 83 Tat Chee Avenue, Kowloon, Hong Kong

## Abstract

Recovering people contours from partial occlusion is a challenging problem in a visual tracking system. Partial occlusions would bring about unreasonable contour changes of the target object. In this paper, a novel method is presented to detect partial occlusion on people contours and recover occluded portions. Unlike other occlusion detection methods, the proposed method is only based on contours, which makes itself more flexible to be extended for further applications. Experiments with synthetic images demonstrate the accuracy of the method for detecting partial occlusions, and experiments on real-world video sequence are also carried out to prove that the method is also good enough to be used to recover target contours.

## 1. Introduction

**Figure 1:**
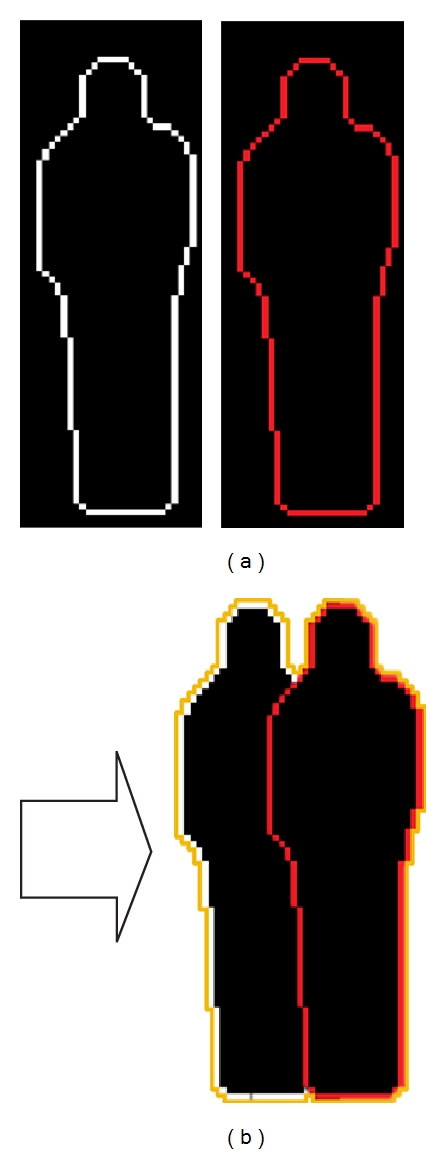
An example of composite contours. (a) is two similar contours of pedestrians. (b) is the composite contours. It is obvious that the contour in (b) is different from that in (a).

In a visual tracking system, the accuracy of object detection and localization usually greatly affects the performance of tracking system and thus it has been one of the key links of the whole system. However, a moving target object is often occluded by background or other moving objects, which makes the target difficult to be detected and localized. Especially when the target is a deformable object [1, 2], it is more difficult to distinguish the occluded parts from the deformable parts [3]. There are a lot of efforts made in detecting and handling the occlusion problem in the literature [4–6]. Most of these methods adopt color [7, 8], appearance [9–11], texture [12], or motion [13] features as clues to detect occlusions. But these methods often tend to fail in detecting and localizing the target when the occluder has the same color, appearance, or texture as the occluded targets or when it has occluded the targets for a long time. However, using contour as a clue can overcome this shortage. For example, as shown in Figure 1, although the contours of occluder and occluded object are completely the same, the overlapped composite contour of two objects is obviously different from that of the individual contour.

In this paper a novel method based on contour is presented to detect the partial occlusions of the target and to recover the complete shape of the target in a tracking system. But the existence of noise and deformation on contour increases the difficulty of occlusion detection. Therefore, the practical method of this study is elaborately developed to avoid the influence of noise and deformation. Our method mainly consists of the following steps. First, the models of shape to be detected will be statistically constructed from several viewpoints. Second, the obtained contours of target are used to match the shape models. During the matching process, a morphing-detecting strategy is introduced to detect the occluded regions. After an initial matching by integral invariants [14], the contours are morphed from the models to the current contours. The partial occlusions can be detected by comparing the times of the break change of contours with a threshold. Third, the recovered shape can be obtained by shape registration (e.g., thin plate spline [15]) after excluding the detected occlusions. There are several advantages of this method: (i) with the help of the morphing process, it can overcome the insufficiency of information when the partial occlusion is detected only by object contours. (ii) The partial occlusion regions are determined by the accumulated evidence during morphing process, which can improve the robustness and accuracy of the results. (iii) A new descriptor insensitive to noise is presented to precisely describe the change of object boundary.

### 1.1. Related Works

Since occlusion is a common but difficult problem in a tracking system, many efforts have been made to solve the problem by using a large variety of features of image [10, 16]. As one of the most popular features, color is often adopted as a clue. But when the tracked objects have similar color to background and other objects, it will cause ambiguity and result in tracking failure. Therefore, some extra assumptions have to be made in some methods that the color of target objects must be distinguished from other objects [7, 8]. Nguyen and Smeulders used appearance model for occluded object tracking. But it can only handle occlusions within a short time [10]. Yilmaz et al. used both the distance among the objects and the change of the object size to detect the occlusions [9]. Although it is an effective detecting strategy, only the coarse location of the occluded parts can be determined, which causes the more complex steps and less accurate result for the recovering of missing object parts. Pan and Hu proposed a method by detecting the nontarget region in ROI to handle the position of the occlusion by using of the spatiotemporal context and motion constraint [11]. This method enhanced the robustness under occlusions. However, the authors acknowledged that it still has some failure modes when the appearances of the moving objects are similar to that of occluder. As a kind of informative feature, texture is also used for occlusion detection. For example, Zhu et al. developed a method for objects detection and occlusion estimation by finding the corresponding parts with similar texture feature and spatial feature in consecutive frames [12]. More recently, Stein and Hebert [13] presented a local detection of occlusion boundaries by using motion as clues in short video clips. But when two objects have the same motions, failure will be inevitable.

Unlike these methods mentioned above, the proposed method uses only the contour of the targets to detect occlusions and recover the exact contour of them. By using the proposed method, one can accurately localize the occluded parts in object contours without the help of any other features of image. Since this method does not need color, texture, and motion information, it will not result in failure when such information is ambiguous. It can also succeed in localizing a target occluded from a long time owing to the ability of recovering target shape directly from object contours.

The remainder of this paper is organized as follows. In Section 2, the building process of target models is described in detail. We give details of occlusions detection and contours recovering in Section 3. Experimental results and conclusions are sketched in Sections 4 and 5, respectively.

## 2. Building Target Models

In this study, our goal is to detect the occlusions on the target contours and then use them to exactly recover the whole target contours in the tracking system; in particular, we focus our efforts on occlusion detection and contour recovering of pedestrians. Since in videos the pedestrians may appear in different viewpoints, there is no uniform contour model which can be simply used to represent the contour exactly. Therefore, three sets of models have been built for representing a pedestrian from three different viewpoints, which are the front, the left, and the right models. These models are most typical and fundamental from which the contours from other viewpoints can be deformed. 

**Figure 2:**
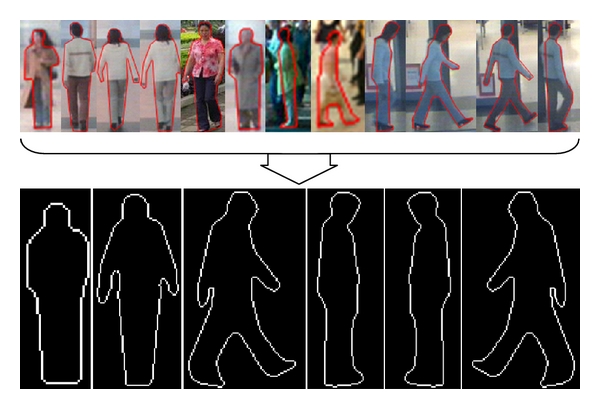
The models of a pedestrian. The first row is the subset of our training set. The second row shows the models. The two on the left are the models from the front viewpoint, the two n the middle are the models from the right viewpoint, and the two on the right are the models from the left viewpoint.

First, we extract pedestrian contours in the training set from three different viewpoints. Each set of three-viewpoint contours has two postures, which are the standing posture and the walking posture, respectively. According to their viewpoints, these contours are aligned, respectively. The average contours are calculated from these aligned contours. The ultimate contours of models are obtained by smoothing these average contours. Because the contours viewed from the back are very similar to those viewed from the front, we assume that the models of them are also the same. The model of the left has the same posture as the model of the right but their directions are opposite. Therefore, three sets of models with six postures are constructed for a pedestrian. As shown in Figure 2, the first row is the subset of the training set and the second row is our six postures of the models.

**Figure 3:**
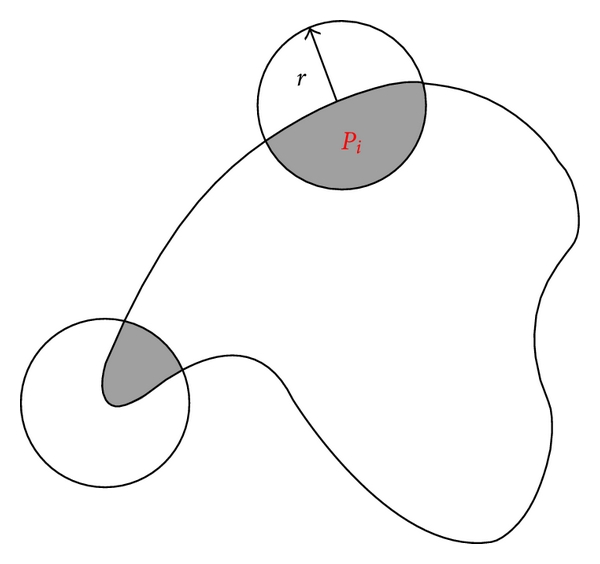
The local area integral invariant. The *r* is the radius of circle, and *P*_*i*_ is the centre. The gray region is the local area integral invariant which is bounded by the circle and object boundary.

Second, we compare a target contour obtained from a tracking system with each of the six models. We match the target contour to six models using the method in [14] to calculate the dissimilarities between the target and each of the six models. The model which has the minimal dissimilarity with the target contour is chosen as the matching model. Manay et al. introduced local area integral invariants to match shapes. A local area integral invariant is defined by the area bounded by a circle and the shape boundary. Because the local area integral invariant is much more robust than other invariants, it can obtain more accurate matching results, even if there exist the deformations on subparts, missing parts, or noise.  Figure 3 shows an example of local area integral invariant. In the proposed method, we adopt normalized local area integral invariant obtained by dividing it by the whole circle area as shape descriptor and use it to compute the dissimilarity between the target contours and models.

## 3. Occlusion Detection

Nowadays, there are many successful methods that can be used to segment the foreground from the background and obtain the target contours. Although these obtained contours may be not the exact object boundaries, the proposed method still can be used to correctly detect the occlusions and recover the target contours. Since we focus on the occlusion detection, to obtain the target contours will not be described in this paper. In this section, we will describe the occlusion detection in detail.

**Figure 4:**
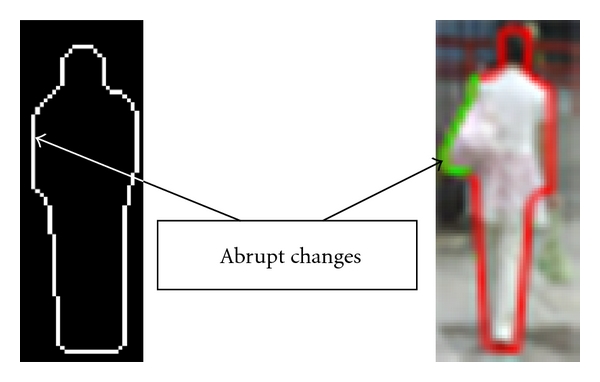
The changes caused by deformation and by occlusion. The red lines mean the gradual changes and the green lines are the abrupt changes.

Given contours of the foreground, whether and where the contours are occluded should be concluded by comparing them with models. It is quite difficult to directly detect the occlusion since the difference between the change of the contours and the occlusions is very ambiguous. But fortunately, inspired by the observation, in general we found that the changes in neighboring parts of a deformed boundary are gradual but the changes caused by occlusions are abrupt, as illustrated by red and green lines in Figure 4. 

Since one part of an object is always connected with its neighbor parts, the change of this part will inevitably affect its neighbors by the action of internal force. The affection is tapering off along with the increasing distances between the changed part and its neighbors. Thus, it causes the gradual changes of contour. But when there are partial occlusions on object contours the gradual changes will be broken and abrupt changes will arise on the object contours of occluded parts relative to those of unoccluded parts. Note that the abrupt change mentioned here means continuously abrupt change, which must be distinguished from the change caused by articulation movement. When articulations move, only the contours around articulations will change, while the other parts will not change. Therefore, the abrupt change caused by articulation movement cannot last and will not be detected as occlusion by our method.

Hence, the occluded parts can be detected by comparing the changes between the model of object contours and corresponding parts of the target, and consequently the location of occlusions can be found according to the detected abrupt changes. An intuitive method is to match the target with the model and directly compute the changes of each pair of corresponding contours to find the abrupt changed parts. However, such a method is crude and sensitive to noise that it will lead to the incorrect results when there are mismatched parts. Therefore, for precisely detecting and localizing the occlusion parts, it needs a more effective and more robust strategy. The proposed method introduces a morphing process in which the model is gradually deformed to the target. At each deforming step, the model is changed slightly, and the influence by mismatching can be eliminated by smoothing deformed contours. And we accumulate the abrupt change times for each part of model during morphing process, which is insensitive to noise. In this way, the proposed method can completely overcome the shortages of the crude method mentioned above.

Our method mainly consists of three stages. Firstly, after the contours of target are obtained by background subtraction (i.e., [17, 18]), the model is sampled as points set and a spring-mass model is constructed to denote a morphing process which deforms the model contour to the target step by step. Secondly, after the intermediate positions of points of the points set are obtained, the changes between two contours, which are obtained from the two adjacent steps, are calculated and then the abrupt changes can be detected accurately. Lastly, the decision of occlusion and its location will be concluded by accumulating evidence.

### 3.1. Construct the Spring-Mass Model

**Figure 5:**
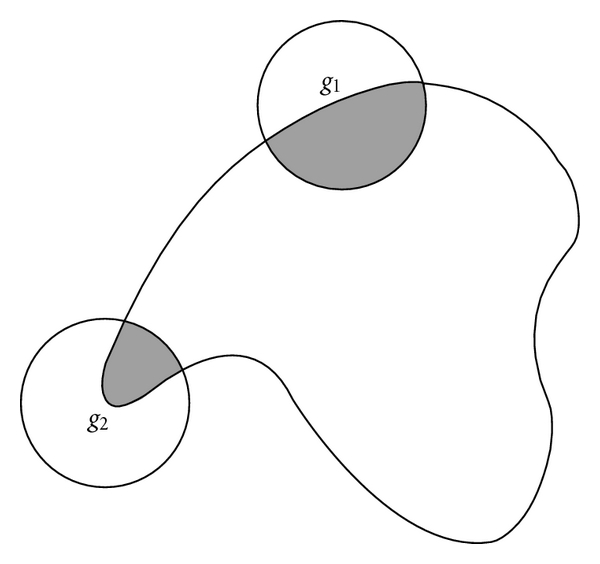
Use LAII to indicate the capability of shape preserving. The LAIIs of all points in segment *g*_1_ are similar, but those of all points in segment *g*_2_ are different. Hence the capability of shape preserving of *g*_1_ is larger than that of *g*_2_.

We firstly sample the matching model to form points set in which the distances between two neighbor points are equal along the contour. Denote the points set as *P* = {*p*_1_, *p*_2_,…, *p*_*n*_}. Each point in *P* is treated as a mass, and the line connecting two adjacent points is regarded as a spring. Then the spring stiffness which is assumed as a constant for each spring needs to be set. We define the term “self-similarity of segment” as the similarity between small neighbors of one point and large neighbors of this point. We then make the following assumption: if the self-similarity of segment of one point is larger, the capability of this point preserving its geometrical shape is stronger. This means that its change is expected to be small when the object contours are deformed. For example, there are two points *p*_1_ and *p*_2_ on two segments of object contours, *g*_1_ and *g*_2_, respectively. As shown in Figure 5, two circles bounding the segments have the same radius. The curvatures of all points on *g*_1_ are similar but those of *g*_2_ are not, which makes the probability of the segment *g*_1_ preserving its geometrical shape higher than that of *g*_2_ when the object contours are deformed. Thus, the “stiffness” of one segment with higher capability of shape preserving is much larger than that of the segments with lower capability of shape preserving. Therefore, the sum of curvatures in the neighborhood of one point can be used to measure this segment “stiffness.” However, the curvature is quite sensitive to noise since it is a kind of derivative of contours. Hence, we introduce the local area integral invariant (LAII) to measure the shape-preserving capability of segment, as shown in Figure 5.

For each point in *P*, *k* LAIIs, corresponding to *k* radiuses, *r*_1_ to *r*_*k*_ with ascending sort, are calculated. The *j*th LAII of the *i*th point is denoted as *A*_*ij*_. Thus the *self-similarity* of segment of shape in the neighborhood of *p*_*i*_ is calculated as follow:

(1) $$C_{i}=\overset{K-1}{\underset{j=1}{\sum }}\left|A_{i,j}-A_{i,j+1}\right|.$$

Then the stiffness of spring between the *i*th and (*i* + 1)th points can be defined as

(2) $$s_{i}=\alpha \left(\frac{C_{i}+C_{i+1}}{2}\right),$$

where *α* is a constant for adjusting the stiffness which can be set to 1 in general. The spring stiffness in different parts of an object is also assumed to be unable to change a lot. Therefore some constraints must be added to avoid the large variation of the stiffness among the adjacent parts. Equation (3) is used to ensure the stiffness changes gradually:

(3) $$s_{i}=\{\begin{array}{cc} s_{i} & \frac{1}{t}\leq \frac{s_{i}}{s_{i-1}}<t,\\ ts_{i} & \frac{s_{i}}{s_{i-1}}<\frac{1}{t},\\ \frac{s_{i}}{t} & \frac{s_{i}}{s_{i-1}}>t,\\ s_{1} & i=1, \end{array}$$

where *t* is a constant used to restrict the maximal variation of stiffness between two adjacent parts (in our experiments, *t* is set to 1.1).

After obtaining these stiffness constants, we then calculate the external force and move the spring-mass model in the next subsection.

### 3.2. Obtain the Intermediate Contours

For moving the spring-mass model, there must be external forces acting on the mass point. Now the method to calculate these external forces is illustrated in detail. After matching the target contours with object shape model by local area integral invariants, each point in point set *P* has a corresponding point in target contours. But there are some mismatching points which can be classified into two categories. In the first category, the mismatching points in the model are corresponding to the occluded parts of the target which make up the pseudocontour of the target. In the second category, the mismatching points belong to the parts of unoccluded contours. The main difference between the two categories is that the mismatching points in the first category are continuous since those points locating in the occluded parts are all mismatched but those in the second category are not. The first category is used as a clue to detect the abrupt changes in our method. But the second category will affect the detected results. Therefore, we employ the thin plate spline to refine the matching result. We deform the model to the target according to the initially matching result. Denote the deformed model as *P*′. The refined matching result can be obtained by finding the nearest corresponding point *q*_*j*_ on the target for each point *p*_*i*_′ on *P*′. After refining, there are only few second category mismatching points. The disturbance of them will be eliminated during the morphing process by slightly smoothing the deformed model. The following equation is defined to calculate the external force acting on the *i*th point on template:

(4) $$f_{i}=\beta ||q_{j}-p_{i}||\left(q_{j}-p_{i}\right),$$

where the magnitude of *f*_*i*_ is determined by *β*||*q*_*j*_ − *p*_*i*_||, and the direction is determined by (*q*_*j*_ − *p*_*i*_). *β* is a constant for all points which controls the magnitude of external force (in our experiments it is set to 0.1).

The next statement will explain the movement principle of the model under the action of the external force. The movement of each point in *P* is determined by three forces: the external force, the internal force produced by the adjacent spring, and the friction force. Mathematically, the principle of movement can be expressed as

(5) $${\vec{F}}_{i}^{\text{ext}}-{\vec{F}}_{i}^{\mathrm{int}}-c{\vec{v}}_{i}=m_{i}{\vec{a}}_{i},$$

where ${\vec{F}}_{i}^{\text{ext}}$ is the external force, calculated by (4), and ${\vec{F}}_{i}^{\mathrm{int}}$ is the internal force which is formulated in (7). The friction force $c{\vec{v}}_{i}$ is proportioned to the velocity of point *p*_*i*_, in which *c* is a constant. Denote *l*_*i*_ as the displacement of *p*_*i*_ and ${\vec{v}}_{i}=d{\vec{l}}_{i}/dt$ as the velocity of point *p*_*i*_. ${\vec{a}}_{i}=d^{2}{\vec{l}}_{i}/dt^{2}$ is the acceleration of this point. Then (5) is converted to the following equation:

(6) $${\vec{F}}_{i}^{\text{ext}}-{\vec{F}}_{i}^{\mathrm{int}}-c\frac{d{\vec{l}}_{i}}{dt_{i}}-m_{i}\frac{d^{2}{\vec{l}}_{i}}{dt_{i}^{2}}=0.$$

In our spring-mass model, each point is connected with two springs. Therefore, the internal force can be formulated as

(7) $$\begin{array}{c} {\vec{F}}_{i1}^{\mathrm{int}}=s_{i-1}\left(\frac{||p_{i}-p_{i-1}||}{||p_{i}^{\prime }-p_{i-1}^{\prime }||}-1\right)\left(p_{i}^{\prime }-p_{i-1}^{\prime }\right),\\ {\vec{F}}_{i2}^{\mathrm{int}}=s_{i}\left(\frac{||p_{i}-p_{i+1}||}{||p_{i}^{\prime }-p_{i+1}^{\prime }||}-1\right)\left(p_{i}^{\prime }-p_{i+1}^{\prime }\right),\\ {\vec{F}}_{i}^{\mathrm{int}}={\vec{F}}_{i1}^{\mathrm{int}}+{\vec{F}}_{i2}^{\mathrm{int}}. \end{array}$$

**Figure 6:**
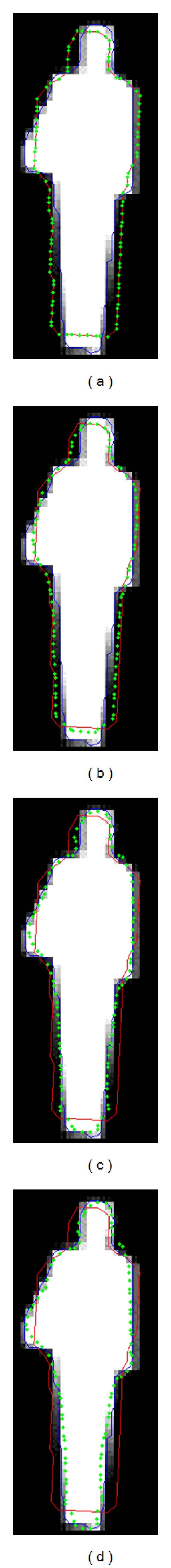
The intermediate contours. The blue shape is the contour of target object, and the red shape is the model. The green points mean the intermediate contours which are moving from model to target. (a) is the initial positions of the intermediate contours. (b) and (c) are the middle positions of the intermediate contours. (d) is the final positions.

Here *p*_*i*−1_′, *p*_*i*_′, and *p*_*i*+1_′ are the instantaneous positions corresponding to *p*_*i*−1_, *p*_i_, and *p*_*i*+1_ at current time, respectively. Equation (6) is an ordinary differential equation. By solving it, the displacement (also new position), velocity, and acceleration of each point at each time can be obtained. The intermediate contours at any time then consist of the positions of these moved points at the corresponding time. Because the spring-mass model will not automatically stop until the sum of external force is equal to zero, we manually stop it when the distance between the moving contours and the target contours reaches its minimum.  Figure 6 illustrated the process. As mentioned above, there are still a few second class mismatching points after refining processing which will influence the morphing process. To deal with it, Gaussian convolution is introduced to smooth the intermediate contours after the new position of each point is obtained.

### 3.3. Detect and Accumulate the Abrupt Changes

**Figure 7:**
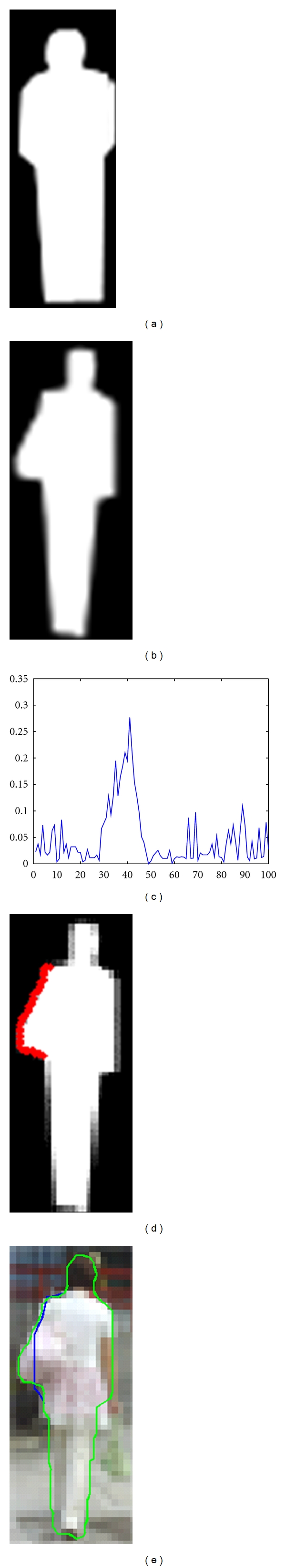
An example of detection and location of occlusion region. (a) and (b) are the template object and the target object, respectively. (c) plots the accumulating evidence of each point. There is obviously one continuous segment with high reliability which is corresponding to the occlusion region plotted by red line in (d). (e) is the result of shape recovering. The green line is the target contours with occlusions and the blue line is the recovered contours. Note that the blue line is visible in occluded parts and is invisible in normal parts as it is superposition with green line.

**Figure 8:**
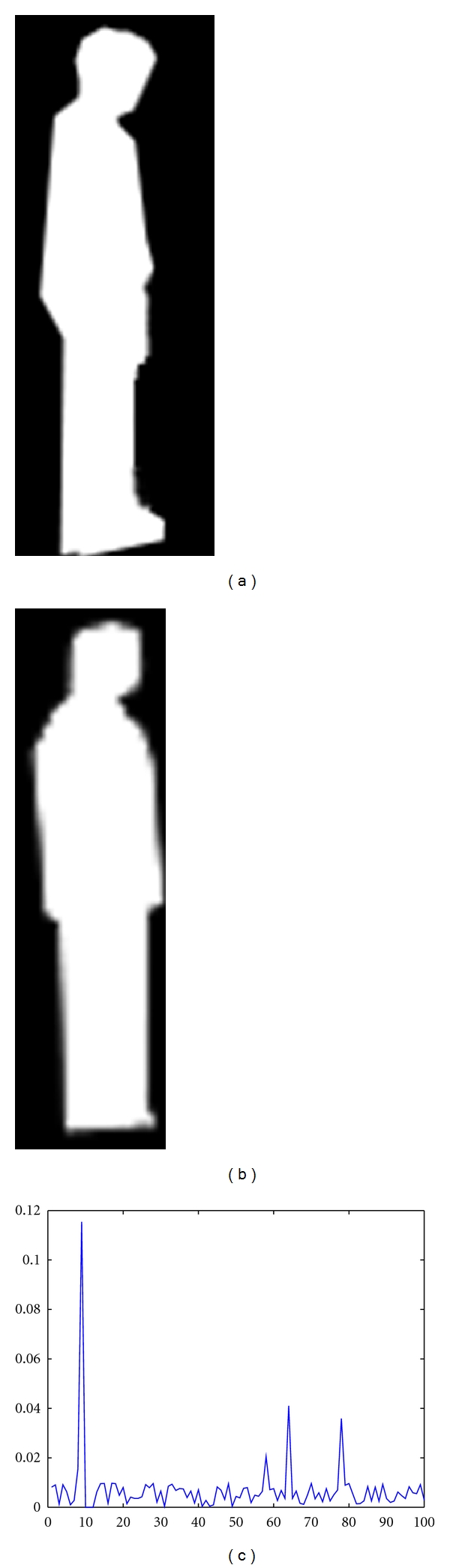
An example of detection and location of occlusion region. (a) and (b) are the template object and the target object, respectively. (c) plots the accumulating evidence of each point. Our method detects the target that has not been occluded since there are no continuous segments with high reliability.

After obtaining the intermediate contours, we then compute *e*_*i*_, the change of the *i*th point between two adjacent intermediate contours, *P*′ and *P*′′:

(8) $$e_{i}=\left|d_{i}^{\prime \prime }-d_{i}^{\prime }\right|\left|\theta_{i}^{\prime \prime }-\theta_{i}^{\prime }\right|,$$

where *d*′ is the distance between *p*_*i*_′ and *p*_*i*+1_′which are the *i*th and (*i* + 1)th points in *P*′. *θ*_*i*_′ stands for the angle between *p*_*i*_′ and *p*_*i*+1_′. *d*_*i*_′′ and *θ*_*i*_′′ are the corresponding distance and angle in *P*′′. Equation (8) measures the change by multiplying the difference of distance and that of angle. Since *e*_*i*_ is computed from the difference of distance and the difference of angle, this kind of measurement is invariant to scale, rotation, and translation. But it may be sensitive to noise. We modify (8) to be more robust, and then we have the following equation:

(9) $$e_{i}^{\prime }=\frac{\sum_{j=i-k}^{i+k}e_{j}\cdot \exp\left(e_{j}-\overset{̅}{e}\right)}{2k+1},$$

where $\overset{̅}{e}=(1/N)\sum_{i=1}^{N}e_{i}$ is the average change of all points. In (9), when *e*_*j*_ is larger (smaller) than $\overset{̅}{e}$, the value of *e*_*j*_ will be enlarged (lessened). Thus, it will amplify the large change and suspend the small change and lead to more robust results to detect the abrupt changes. And using the average change of 2*k* + 1 points, which are *k* former and latter points of the *i*th point, respectively, can further improve its robustness. There are *h* + 1*e*_*i*_′ computed from the model contours, *h* intermediate contours, and one target contour. And then the amount of abrupt change times for each point is accumulated:

(10) $$v_{i}=\overset{h+1}{\underset{i=1}{\sum }}f\left(e_{i}^{\prime }\right),$$

where *f*(*e*_*i*_′) is an indicator function of the abrupt change:

(11) $$f\left(e_{i}^{\prime }\right)=\{\begin{array}{cc} 1 & e_{i}^{\prime }-T_{s}\geq 0,\\ 0 & e_{i}^{\prime }-T_{s}<0, \end{array}$$

where *T*_*s*_ is a threshold to indicate that the change is abrupt or gradual (in our experiments *T*_*s*_ is set to *μ* + 2*σ*, where *μ* is the average of *e*_*i*_′ and *σ* is the variance of it [19]). If *v*_*i*_ is larger than a threshold *T*_*v*_ (in our experiments *T*_*v*_ is set to 2*μ*, and *μ* is the average of *v*_*i*_), the point *p*_*i*_ may be in an occluded region. Note that the occluded region must have a certain extent. Therefore, if one point is in an occluded region, a certain number of neighbor points must also be in the same occluded region. Thus, we can set another threshold *T*_*n*_ (in our experiments *T*_*n*_ is set to *n*/10, where *n* is the number of points in template point set) which indicates the least number of points in an occluded region. Finally, if the amount of sequence points all of which *v*_*i*_ are large than *T*_*v*_ is not less than *T*_*n*_, the sequence points indicate an occluded region (see Figure 7 as an example). If there is not any sequence points all of which *v*_*i*_ are larger than *T*_*v*_ is larger than *T*_*n*_, the target contour is an deformed contours without occlusions. An example is illustrated in Figure 8. 

### 3.4. Recover Target Contours

After detecting the occlusion regions, the target contour then can be recovered conveniently. The parts of model corresponding to the parts of target contours detected as occluded regions are excluded at first. We then sample a certain number of points from the target contours excluded occlusions and the corresponding points in the shape model as anchor points. The thin plate spline is employed to warp the shape model to target contours.  Figure 7(e) illustrated an example of contours recovering. For more accurately recovering, we can use more complex methods [20, 21]. For example, the shape context [22] is an excellent shape register method based on a sample point set which can increase the accuracy of the recovered shape. The integral invariant is also an excellent shape matching and register method, but in our method we do not employ it to recover the target contours since it needs the closed contours.

## 4. Experiments

**Figure 9:**
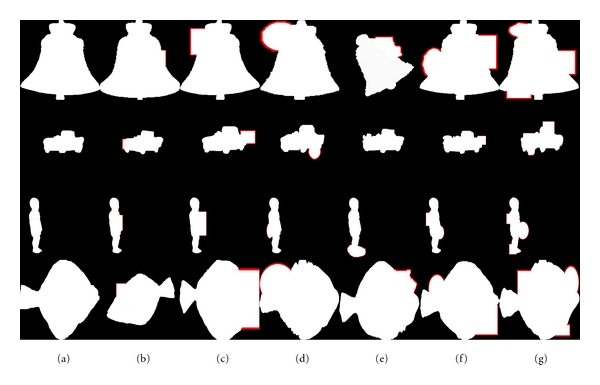
Some examples of occluded images based on MPEG-7 dataset. (a) is selected as the template image. (b), (c), (d), and (e) are the occluded images by single object under different occlusion region. (f) is the occluded image by two objects. Images in (g) are occluded by three objects. The red line indicates the occlusion region.

We firstly evaluate the performance of occlusion detection method on an occluded version of MPEG-7 dataset [23] in which we added occlusion regions of different sizes and numbers to the images (Figure 9 shows some examples). 

**Figure 10:**
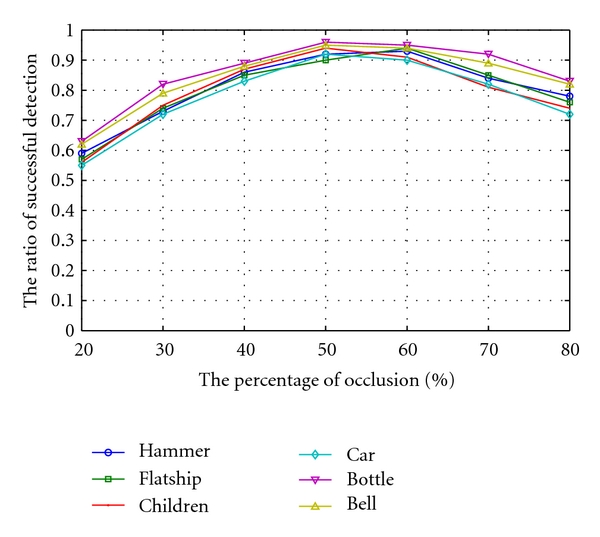
The average ratio of successful detection of occlusion region of each class image.

 We select six classes of images as testing images, which are the bell, the car, the children, the flatship, the bottle, and the hammer. From each class one image is chosen as the template (see Figure 9(a)), and the other images are occluded by shapes of different sizes and numbers. For each image in a class, the object boundary is occluded by one rectangle, ellipse, and random shape, respectively, and the occlusion region in contour ranges from 20 to 80 percent (see Figures 9(b), 9(c), 9(d), and 9(e)). Each image is also occluded by two or three shapes simultaneously. It brings about multiocclusion regions, and the amount of occlusion contour also ranged from 20 to 80 percent (see Figures 9(f) and 9(g)). After obtaining the occlusion parts detected by our method, we compare them with the actual occlusion regions. If the overlapped parts are not less than 90 percent and not more than 110 percent, we believe the detection is successful. Otherwise the detection fails.  Figure 10 shows the average ratio of successful detection of all six classes' images. When the occlusion region is too small, the ratio of successful detection is low since it is rather ambiguous between the small-scale partial occlusions and deformation. But with the occlusion regions increasing, the ratio of successful detection is also mounting up until 60 percent. When the occlusion region is more than 60 percent, the successful ratio will decrease from the highest ratio since the normal boundary is too small to distinguish the occlusion part. But we can regard the occluder as the model which is occluded by target, by which the new occlusion region is less than 60 percentage and the high ratio of successful detection can be obtained. 

**Figure 11:**
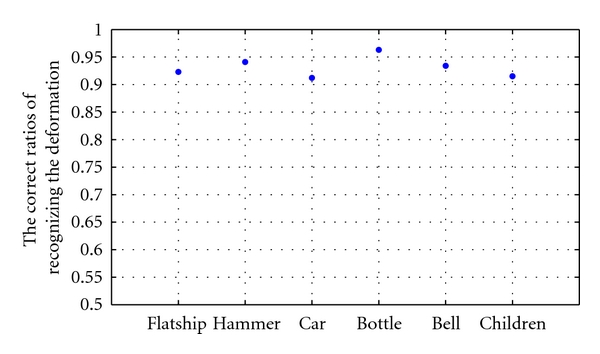
The correct ratios of recognizing the deformation.

Because it is quite easy to confuse the partial occluded contours with deformed contours, the performance of the proposed method to correctly recognize the deformed object contours must also be evaluated. By choosing one image as model in a class, our method is used to detect other normal images in the same class. If there is no occlusion region after the detection, it shows that our method can correctly recognize the deformed object boundary.  Figure 11 illustrates these correct ratios of the six classes' objects.

It can be seen from Figure 11 that the bottle class has the highest recognition ratio since its shape is the simplest and the degree of deformation is the smallest. Both the children class and car class have the relative lower recognition ratio since their shapes are more complicated. Note that in Figure 10, these two classes also gain the relative lower ratios of successful detection. But a complex object can be decomposed into the combination of simple object by some shape decomposition method (i.e., [24, 25]) to improve the performance of our method.

**Figure 12:**
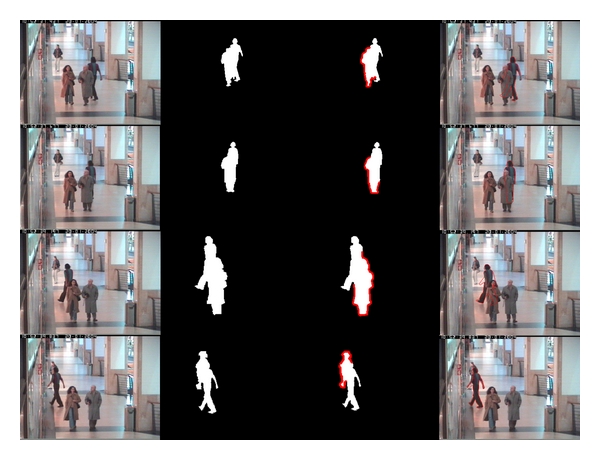
Examples of occlusion detection and target shape recovering in real-world video sequences. The frame numbers from top to bottom are 1983, 1989, 2027, and 2043, respectively. The columns from left to right are original frames, foreground contours, results of occlusion detection, and results of shape recovering, respectively. In the second column, we have eliminated other foreground contours which are not connected to our target for clearer display. Note that the results of occlusion detection are quite exact but the result of shape recovering in the third row is failure.

Next, we apply the proposed method to detect occlusions and recover target shape in a tracking system.  Figure 12 shows the occlusion detection results and contours recovering results of the left viewpoint model, respectively. In Figure 12, each row corresponds to one frame in real-world video. From left to right, the columns are the original images, foreground contours around the target, the occlusion contours detected by the proposed method, and the recovered contours of target. We extracted the foreground by using background subtraction and neglected those too small patches. All six models of pedestrian are matched to the contours of each disconnected foreground region for computing the dissimilarities. The model corresponding to the minimal dissimilarity is chosen for detecting the occlusions and recovering target contours. If the minimal dissimilarity is larger than a threshold, we believe that this foreground region does not contain the target and ignore it before we match the next foreground region.

As shown in Figure 12, the results of occlusion detection are accurate since the percentage of occluded parts is just between 30% and 70%. This is in line with the previous experiments. The results of shape recovering are not as accurate as occlusion detection since we just use the TPS method to recover target shapes. To improve this, one can employ some more complicated methods (i.e., shape context [22]).

## 5. Conclusions and Discussions

The main contribution of this paper is that a novel method is presented to detect partial occlusion based on target contours. With the help of this method, satisfactory performances in both synthetic and real-world image sequences are achieved for object tracking and target contours recovering. In our method contour matching is taken as a morphing process from which sufficient information is obtained to detect the partially occluded regions and the influence of mismatching and noise can also be overcome. The method only needs object contours, which can be used in the situation where color, texture, and motion information cannot be obtained exactly.

However, there are still some further works for improving the performance of algorithm. As mentioned in Section 4, a relatively low detection ratio was observed when the occlusion region is small due to the extreme ambiguity between the deformed contours and small-scale occluded contours. When the object to be detected is complex, the method needs to be further enhanced, for example, by employing some shape decomposition methods to decompose a contour to a certain number of simple objects and then detect them separately. During the processing of target contours recovering, we simply use the thin-plate spline to register the model to target. To improve this, some more complex methods can be adopted.
